# *Purshia plicata* Triggers and Regulates Proteins Related to Apoptosis in HeLa Cancer Cells

**DOI:** 10.3390/plants10122559

**Published:** 2021-11-23

**Authors:** Patricia Álvarez-Ortiz, Juan Ascacio-Valdés, Ileana Vera-Reyes, Cecilia Esparza-González, Raúl Rodríguez-Herrera, Mauricio Salinas-Santander, Mayela del Ángel-Martínez, Antonio Morlett-Chávez

**Affiliations:** 1 Laboratory of Molecular Biology, Chemistry School, Autonomous University of Coahuila, Saltillo 25280, Mexico; patriciaelizabethalvarezortiz@hotmail.com; 2 Bioprocesses and Bioproducts Research Group and Laboratory of Molecular Biology, Food Research Department, Chemistry School, Autonomous University of Coahuila, Saltillo 25280, Mexico; alberto_ascaciovaldes@uadec.edu.mx (J.A.-V.); raul.rodriguez@uadec.edu.mx (R.R.-H.); 3 Proteomics Laboratory, Agricultural Plant Science and Biotechnology, Research Center for Applied Chemistry, Blvd. Enrique Reyna 140, Saltillo 25294, Mexico; ileana.vera@ciqa.edu.mx; 4 Laboratory of Histology, Dentistry School, Autonomous University of Coahuila, Saltillo 25125, Mexico; sceciliaesparza@gmail.com; 5 Laboratory of Molecular Biology, Health Research Department, Medicine School, Autonomous University of Coahuila, Saltillo 25000, Mexico; mauriciosalinas@uadec.edu.mx (M.S.-S.); mayela_30@yahoo.com.mx (M.d.Á.-M.); 6 Clinical Laboratory Department, General Hospital No. 2, Mexican Institute of Social Security, Saltillo 25017, Mexico

**Keywords:** *P. plicata*, apoptosis, HeLa, bio-compounds

## Abstract

Cervical cancer represents a public health problem, develops resistance to traditional therapies and cost-of-treatment is high. These disadvantages have led to the search for alternative bioactive-compound-based therapies. Said bioactive compounds include phenolic compounds, flavonoids, and tannins. The present study aimed to evaluate the therapeutic effect of a *P. plicata* extract on the HeLa cell line. Viability and apoptosis assays were run on the two cell lines treated with the extract. The peptides, up- and down-expressed in both cell lines, were identified by PDQuest analysis software and high-performance liquid chromatography/mass spectrometry/mass spectrometry (HPLC/MS/MS). Our results show that a 500 mg/L treatment deregulated cell viability, with different apoptotic morphologies observed which are associated with the presence of bio-compounds, which up- and down-regulated the peptides. In conclusion, *P. plicata* regulates proteins associated with apoptosis in HeLa cancer cells.

## 1. Introduction

In Mexico, as in many countries around the world, cervical cancer (CC) remains a significant health problem [1,2,3]. In sexually active women, CC occupies third or fourth place in the table of the most commonly diagnosed illnesses and causes of death on a global level [1,4]; a statistical variation is evident depending on human papillomavirus (HPV) infection, sex steroid hormones, and environmental and genetic factors [2,5]. All of these factors may contribute to the high incidence of CC, diagnosis at advanced stages, and ineffective treatment [4]. Moreover, the correspondence between these factors and the early stages of carcinogenesis is currently unknown [5]. Current CC treatments show low efficacy and specificity, high treatment costs, relapse in patients whose condition had improved, drug resistance, and decreased quality of life [3,6,7,8]. Traditional medicine may provide an opportunity to improve CC treatment and prognoses. Pertaining to the family *Rosaceae*, *Purshia plicata*, also known as Rosa de Castilla, is endemic to the Chihuahua Desert [9] and is used in traditional Mexican medicine for the treatment of kidney and stomach diseases.

The pharmaceutical properties of plants depend on the quantity and quality of bioactive/phytochemical compounds [10]. Prior research has described some fruits or plants growing in the northeast of Mexico as alternative sources of bio-compounds [11]. While Rosa de Castilla presented a high quantity of bioactive/phytochemical compounds, there is little information on the properties, type, number, and quality of its polyphenol compounds. Early research identified catechin, quercetin (Qu), and ellagic acid as compounds potentially present in Rosa de Castilla [12]. Kaempferol and Qu exhibit potential therapeutic properties against tumour cell lines, but not non-transformed cells, and can enhance apoptosis, induce cell cycle arrest, and reduce reactive oxygen species (ROS), particularly in tumour cells or chemo-resistant cells [3,7,8,13]. Other polyphenols, such as resveratrol, catechin, and epigallocatechin-3-gallate, decreased the proliferation of some cancer cell lines [14], and some of them may induce apoptosis [15] and suppress angiogenesis and invasiveness as well [16,17,18].

Although there is plenty of information on the effects of bio-compounds and phytochemicals on cancer cells, gaps remain in the literature about their mechanisms against cancer. For example, alternative strategies for improving the effectiveness of bio-compounds and overcoming drug resistance are required [19]. Given that only a few of these bio-compounds have therapeutic potential against cancer, these therapies must be evaluated via in vitro antiproliferation assays using cultures containing both tumour and non-tumour cells, as well as different cell models [20]. The present study evaluates the protective effect of a *P. plicata* extract and describes the capacity to trigger or block the oncogenes that participate in HeLa cancer cells. Additionally, we demonstrated the expression of peptides related to the apoptotic process in treated cells. A mix of polyphenols was obtained from *P. plicata* and evaluated in terms of its effect on HeLa and fibroblast 3T3 cell lines. The viability and apoptosis assays were run on both cell lines treated with the extract, while the peptide profile was analysed for both cell lines, with the peptides identified via PDQuest and HPLC/MS/MS. Our results showed that a 500 mg/L treatment was required to increase cell viability to 55%, while morphological changes were observed in HeLa cells exposed to a 500 mg/L treatment, as were observed apoptotic nuclei and bodies. Peptides related to those that trigger apoptosis were also identified. All experiments were conducted in triplicate, with T student statistical analysis showing α < 0.05 for each experiment.

## 2. Materials and Methods

### 2.1. Purshia plicata Extract

The plant was collected and identified by a botanist of the Antonio Narro Autonomous Agrarian University. The mix of more than 25 polyphenols was considered as an extract for this paper. The extract was donated by the group of Ascacio-Valdés and co-workers. The extract was obtained through assisted fermentation. The recovery of the extract was performed with distilled water and was partially purified using amberlite. The same group identified the composition of the extract by high-performance liquid chromatography (HPLC). The most abundant compounds identified in the extract were kaempferol 3,7-O-diglucoside, procyanidin trimer C1, and ellagic acid (Table 1) [12]. Some of the polyphenols found in the extract are listed in Table 1 as well as their families (Table 1).

### 2.2. Cell Culture

Human cervical cancer cells and mouse fibroblast cells (HeLa and 3T3 cell lines, respectively) were obtained from the American Type Culture Collection (ATCC), cultured in A-DMEM medium (Advanced Gibco Dulbecco’s Modified Eagle Medium), and supplemented with 10% foetal bovine serum (FBS, BIOPIONWWE), 4 mM L-arginine, and 1% penicillin–streptomycin. The cells were incubated at 37 °C in a humidified atmosphere with 5% CO_2_. The 3T3 cell line was used as a control as it was not a tumoral cell line.

### 2.3. Cell Viability

#### MTT Assay

The cells (7.5 × 10^3^ cells/well) were seeded on 96-well plates and treated with different concentrations of the extract (62.6, 125, 250, 500, and 1000 mg/L) for 24 h. The concentrations were selected via a prior screening process (data not shown). After incubation, 20 μL MTT (3-[4,5-dimethylthiazol-2-yl]-2,5 diphenyl tetrazolium bromide) was added for 4 h at 37 °C, with the MTT then replaced by 100 μL dimethyl sulfoxide (DMSO) in order to dissolve the formazan crystals. The absorbance of the mixture was measured at 575 nm using a microplate reader [21].

### 2.4. Cytotoxic Effect

#### LDH Assay

The evaluation was conducted using Cayman’s Lactate dehydrogenase (LDH) Cytotoxicity Assay Kit (Cayman Chemical, Ann Arbor, MI, USA), wherein the same number of cells were seeded on 96-well plates containing 200 μL culture medium. After 24 h, 20 μL triton X-100 was added to three wells (positive control), 20 μL assay buffer was added to three wells (spontaneous release), and 20 μL LDH positive control was added to another three wells, with different concentrations of extract then added to the appropriate wells. After 24 h of incubation, the plate was centrifuged and 100 μL supernatant was transferred from the wells to a new 96-well assay plate, with 100 μL LDH reaction solution then added to each well. The plate was incubated and shaken gently for 30 min. The absorbance was recorded at 490 nm [22].

### 2.5. Characterization of Cell Morphologic Changes and Comet Assay (Single Cell Gel Electrophoresis)

HeLa and 3T3 cells (7.5 × 10^3^ cells/well) were seeded on 96-well plates and exposed to different concentrations (250, 500 and 1000 mg/L) of *P. plicata* extract for 24 h. After incubation, the cellular morphology was observed using a Dino-Eye Microscope Eye-piece camera in the Dino Capture 2.0 program, while the DNA fragmentation was evaluated via comet assay. After treatment with the extract (250, 500, and 1000 mg/L) for 24 h, the cells were harvested and suspended in cold phosphate buffered saline (PBS). The suspension was combined with agarose (1:10) and, after solidification, the plate was immersed in lysis buffer (OxiSelect) for two hours. The plate was placed in an electrophoresis chamber with electrophoresis buffer tris-acetate-ethylenediaminetriacetic acid (EDTA) (TAE) (40 mM Tris-acetate, pH 8.5, 2 mM EDTA). The assay was conducted for 20 min in an electric field at 300 mA. Subsequently, the plate was washed with distilled water, with Vista Green DNA dye (Oxiselect) then added to each well. A fluorescence microscope was used to analyse the comets of the cells [23,24].

### 2.6. Evaluation of Protein Expression

HeLa cells (90% confluence) exposed to 500 mg/L of *P. plicata* extract were washed with 1 mL PBS and resuspended. The suspension was centrifuged at 2500 rpm for 10 min and the supernatant was discarded, and the pellet was mixed with extraction buffer and centrifuged at 10,000 rpm to recover the supernatant. The addition of acetone (*V*/*V* 1:1) and posterior centrifuge at 13,000 rpm for 5 min led to the obtaining of a plug of protein, which was posteriorly resuspended on 100 µL of MilliQ water. Proteins were quantified by the Bradford method.

To assess total protein expression, one-dimensional sodium dodecyl sulfate-polyacrylamide gel electrophoresis (SDS-PAGE) was performed on resuspended proteins. A 5–12% polyacrylamide gel of 1 mm thickness was used. Protein samples were loaded (15 µL sample with 5 µL denaturalizing buffer) on a gel; electrophoresis was achieved with an 80 V electric field for 2 h. The gel was stained with Coomassie Brilliant Blue G-250 [25]. Additionally, to determine the expression of specific peptides, two-dimensional electrophoresis was carried out. Protein samples (>169 µg) from treated cells were applied for isoelectric focusing (IEF) on Protean IEF cell (Bio-Rad) using the ReadyStrips immobilized pH gradient (IPG) strips, 7 cm pH 3–10. The IPG strips were rehydrated with sample and placed into a tray channel and covered with mineral oil at 250 V for 16 h. The first dimension was carried out on the IEF chamber at 50 °C for 5 h. The two-dimension electrophoresis was performed on the strips. Briefly, the strips were washed with 1 mL of equilibration buffer and placed over a 12% polyacrylamide gel previously prepared; finally, 400 µL of agarose was added, and a 100 V electric field was used for 1 h. The gel was stained with Coomassie Brilliant Blue G-250. Each spot of two-dimensional electrophoresis gel was used. Gels were unstained with 50% acetonitrile and 0.1% trifluoroacetic acid solutions. Reduction of the protein sample was conducted using dithiothreitol solution and iodoacetamide to continue the alquilating process. Digestion was conducted using trypsin (10 µL, 12.5 ng/µL) and 50 mM NH_4_HCO_3_. Peptides were obtained on a Centri Vap. Mass fingerprinting was used to identify proteins.

Matrix-Assisted Laser Desorption/Ionization-fingerprinting-mass spectrometry/mass spectrometry (MALDI-fingerprinting-MS/MS) was conducted. Protein expression was analysed with BioTools 3.2 (Bruker Daltonics, Bremen, Alemania) in combination with Mascot algorithm (2.6.2.1 version) against the NCBIprot protein sequence database [26,27].

## 3. Results

### 3.1. Cell Viability

**Figure 1:**
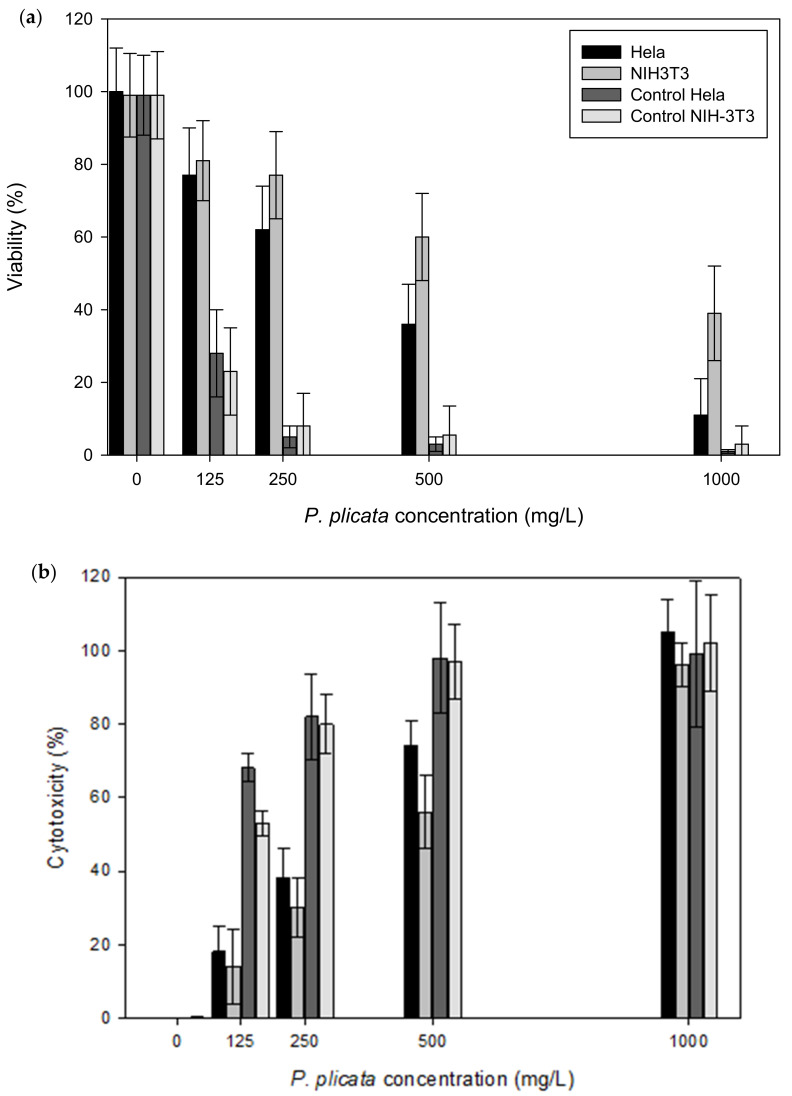
Cell viability and cytotoxic effect. (**a**) The HeLa cancer cells and NIH-3T3 cells were treated with different concentrations of *P. plicata* for 24 h. An amount of 500 mg/L of *P. plicata* extract was required to decrease HeLa cancer cell viability to 45–50%. (**b**) The HeLa cancer cells showed more cytotoxic effects than the NIH-3T3 cells under the same treatment (500 mg/L for 24 h). The HeLa and NIH-3T3 cells were exposed to 10 mg cisplatin for 24 h. The statistical analysis showed α ≤ 0.05.

Based on our results, 500 mg/L of *P. plicata* extract was required to decrease the viability of HeLa cells by 55% in 24 h (Figure 1). Under these same conditions, we observed that the viability of the 3T3 cells decreased by almost 25% in 24 h; this indicates that the extract is more cytotoxic for tumoral cells than for normal cells. To confirm these results, we evaluated the cytotoxic effect of *P. plicata* extract on HeLa cells via LDH assay, with the assays showing a cytotoxic effect of close to 80% on HeLa cells in 24 h (Figure 1) and a minor cytotoxic effect on 3T3 cells exposed to 500 mg/L *P. plicata* extract in 24 h.

### 3.2. DNA Fragmentation in HeLa Cell Induced by P. plicata Extract

**Figure 2:**
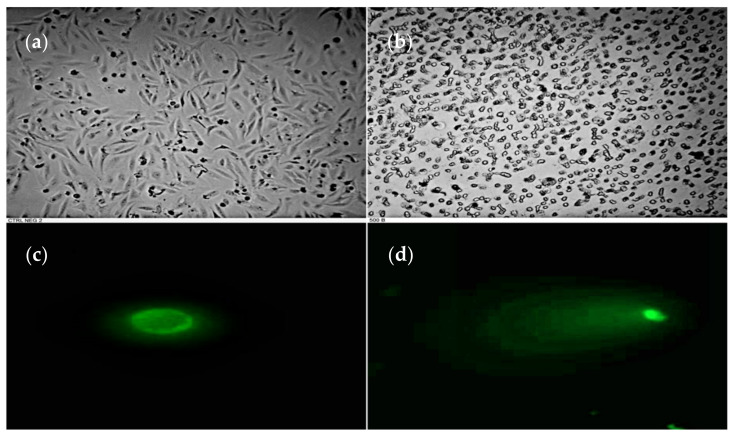
Morphological changes and DNA fragmentation. (**a**) Shows HeLa cancer cells without treatment, with the cells exhibiting a normal morphology; (**b**) shows HeLa cancer cells treated with 500 mg/L *P. plicata* extract for 24 h, with the HeLa cancer cells displaying apoptotic cells. (**c**) Shows a HeLa cancer cell without treatment, which presented a nucleus without DNA fragmentation. (**d**) Shows a HeLa cancer cell after treatment, with the cell’s ‘comet’ clearly visible, thus indicating the presence of an apoptotic nucleus.

Figure 2 shows the evident morphological changes in the HeLa cells treated with 500 mg/L of extracts in comparison with the normal morphology observed for the fibroblast cell line. Specifically, greater evidence of condensation and fragmentation of the nuclei was observed in the HeLa cells, while the 3T3 cell line showed minor damage with the above-mentioned treatment (Figure 2). The comet assay was used to evaluate the DNA fragmentation in the HeLa cells treated with different concentrations of the extract of interest, with the results obtained showing that the cancer cell line exposed to 500 mg/L extract underwent apoptotic changes.

### 3.3. HeLa Cell Peptides Related to the Apoptotic Process

**Figure 3:**
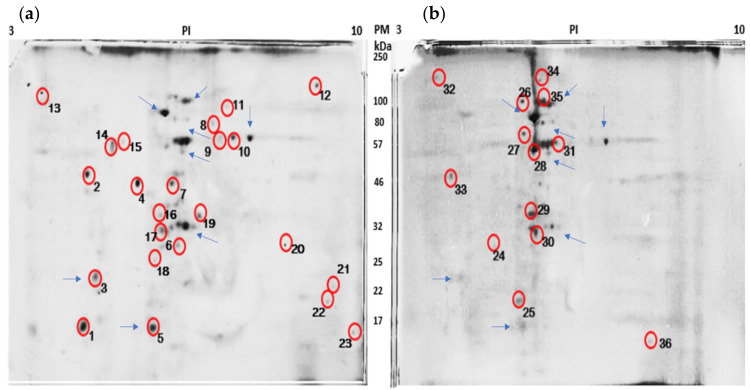
Proteome of HeLa cancer cells. The figure shows two gels containing proteins extracted from the HeLa cancer cells: (**a**) HeLa sample without treatment and (**b**) HeLa sample treated with 500 mg/L extract. Blue arrows indicate anchor proteins. Red circles indicate different proteins.

**Figure 4:**
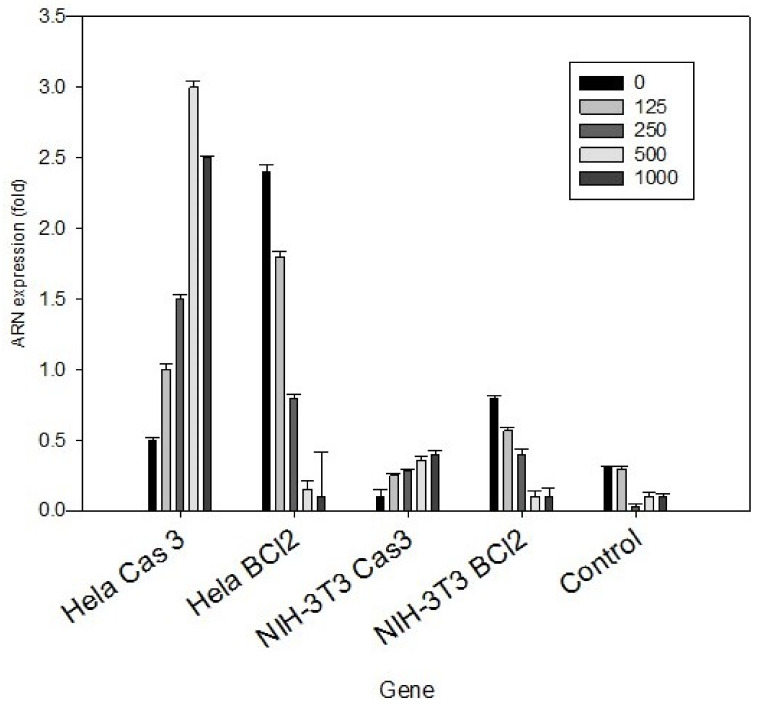
ARN expression of HeLa cancer cell. The ARN of the HeLa cancer cell was analysed by amplifying caspase 3 and Bcl-2. HeLa cancer cell treated with 500 mg/L for 24 h increased caspase 3 expression and decreased Bcl-2 expression, at the same concentration. T-student analysis shows α ≤ 0.05.

In order to confirm the apoptotic process previously described, we analysed the protein expression in HeLa cells exposed to 500 mg/L *P. plicata* extract, with the selection then carried out of the twenty peptides solely expressed in the treated cancer cells (Figure 3). The down-expressed peptide fingerprints observed corresponded to B-cell lymphoma (Bcl2), while the up-regulated fingerprints corresponded to 70-kDa heat shock protein (HSP70), cytochrome C, p53, dynamin-1-like protein, apoptotic protease-activating factor 1 (APAF 1), and caspases, all of which are related to the apoptotic process (Table 2). To confirm previous results, we analysed the ARN expression and demonstrated the increased expression of caspase 3, and BcL-2 expression was decreased with treatment too (Figure 4).

## 4. Discussion

The present research tested the cytotoxic and apoptotic effects in HeLa and fibroblast 3T3 cells exposed to biocompounds, finding that 500 mg/L of Rosa de Castilla extract was required to achieve a HeLa cell viability of 55%. Our findings coincide with those reported previously, in which ethanol extract of moringa peregrina achieved an IC_50_ of 756.13 mg/L in the same cells [1]. Moreover, an ethanolic extract of *Butia odorata* was evaluated on three different cervical cancer cell lines (HeLa, SiHa, and C33a) and a murine fibroblast cell (L929). These same authors noted that the IC_50_ was 528, 412, 1000, and >1000 µg/mL for SiHa, C33a, HeLa, and L929, respectively [28]. Singh et al. (2017) reported that 179.3 µg/mL of methanolic extract of *Bidens pilosa* was required to decrease HeLa cell viability [10]. However, another evaluation reported that HeLa viable cell levels decreased after treatment with 5.67 µg/mL of the hexane partition of *Annona crassiflora* Mart [4]. A flavone pair, apigenin and luteolin, at 40 µmol/L has been observed to have a cytotoxic effect on HeLa cells [29]. Lewinska et al. (2014) estimated IC_50_ at 8 µM in HeLa cancer cells treated with curcumin for 24 h [30].

We observed that the *P. plicata* extract induced significant changes in HeLa cancer cell morphology after 24-h treatment (Figure 2). However, under the same treatment conditions, 3T3 non-cancer cells presented scarcely any morphological changes (Figure 2). There is evidence of multinucleated, flattened, and enlarged cells after the application of curcumin treatment on HeLa cancer cells [30], and HeLa cancer cells treated with apigenin and luteolin for 24 h showed apoptotic morphology, such as the condensation and fragmentation of nuclei [29]. Li et al. (2017) also reported the increased level of both apoptotic bodies and nuclear condensation in HeLa cells treated with resveratrol 20 mM/48 h [31]. Furthermore, a chloroform fraction taken from an ethanolic extract of *M. peregrina* (CFEE) has been reported to induce apoptosis and, thus, inhibit cell growth in HeLa [1]. *Annona muricata* Linn Leaf induces the formation of apoptotic bodies in HeLa cancer cells [32], while TUNEL assays have shown that the flavonoids of *Carya cathayensis* Sargent may inhibit growth and induce apoptosis in HeLa cells [33]. Apoptosis is a complex process, involving a cascade of reactions and multiple genes, that results in the fragmentation and nuclear condensation of DNA.

Proteomic analysis has shown that *P. plicata* extracts regulate the expression of proteins related to the apoptotic process via caspases. The mechanism that induces and triggers apoptosis, as mediated by active compounds, remains only slightly elucidated. Prior reports have demonstrated that active compounds trigger p53 activation, increasing Bax protein levels and decreasing Bcl-2 levels. Prior to the activation of p53 and Bax, cytochrome C binds to APAF-1, after which this complex activates caspases 3, 8 and 9 [31]. In this context, we identified cytochrome C, apoptosis activation factor 1, and caspase 8, all of which were up-expressed in the HeLa cancer cells treated with *P. plicata*. Moreover, the present study identified the cAMP response element-binding protein (CREB)-binding protein (CBP) or its closely related homolog, p300. In the absence of bio-compounds, extracellular signal-regulated kinase ½ (ERK1/2) phosphorylates CREB and up-regulates Bcl-2 and Bcl-X; a pathway which deregulates apoptosis [34]. The phosphorylation of CREB enabled it to bind to BCL9 and b-catenin, while the over-activation of this protein complex is essential for the activation of the aberrant Wnt pathway [35,36]. Furthermore, CREB is capable of activating the protein kinase B (PKB or AKT) and the serine-threonine kinase mammalian target of rapamycin (mTOR), wherein AKT begins to activate the oncogene targets RAS, mitogen/extracellular-signal-regulated kinase (MEK), and extracellular-signal-regulated kinases (ERK). Moreover, RAS induces the activation of phosphatidylinositol 3-kinase (PI3K)/AKT/mTOR [37]. Previous reports have noted that curcumin was the first cell-permeable inhibitor of p300/CREB [38], with further research indicating that curcumin may suppress the RAS/RAF/MEK/ERK cascade and the PI3K/AKT/mTOR-signalling pathway [16,37,39]. Naoi et al. (2019) described that compounds such as epigallocatechin-3-gallate (EGCG) and quercetin up-regulate the ERK/CREB pathway [34].

The overexpression of BCL-6 induces proliferation, migration, and invasion in ovarian cancer cell lines. However, the BCL-6 corepressor (BCOR) enhances the inhibition of BCL-6 in tumour cells. While the present study found the overexpression of BCOR in HeLa cancer cells, we were unable to confirm, via Western blot, the level of BCOR expression. In human endometrial stromal cells, resveratrol has been found to modulate the retinoic acid (RA) pathway, while BTG2 may be downregulated in HeLa cells exposed to *P. plicata*. Resveratrol downregulates the cellular retinoic acid-binding protein 2 (CRABP2) and RA receptor a (RARa) and suppresses the RA-responsive nuclear receptor (PPARβ/δ). Furthermore, RAR regulates the B-cell translocation gene 2 (BTG2), while the downregulation of BTG2 reduces cyclin 1 levels, inhibiting, as a result, G1-S progression [40]. Retinoblastoma (Rb) is another protein that regulates the pass through G1 into the S phase [41]. In a prior study, Riahi-Chebbi et al. (2019) reported that kaempferol treatment in colon cancer cells decreased the expression of RB phosphorylation and cyclin-dependent kinase (cdc2), and enhanced p53 and p-53 phosphorylation [19]. The present study showed the upregulation of Rb and p53 mediated by several polyphenols found in the extract.

## 5. Conclusions

The results of this investigation provide evidence about the cytotoxic effect of *Purshia plicata* extract against HeLa cancer cells. The mechanism of biological activity was DNA fragmentation with evidence of apoptotic processes with the expression of genes and proteins related to the process.

## Figures and Tables

**Table 1:** Example of some compounds found in *P. plicata* extract [12].

ID	Compounds	Family
1	Kaempferol	Flavanols
2	Kaempferol	Flavanols
3	Quercetina	Flavanols
4	Catechin	Catechins
5	Kaempferol	Flavanols
6	Epicatechin	Catechins
7	Quercetin	Flavanols
8	Ellagic acid	
9	Gallic acid	Hydroxybenzoic acids
10	Apigenin	Flavones

**Table 2:** Peptide fingerprint in HeLa cells.

ID	Protein	Sequence
1	Cytochrome C	VLGPDRVLGPDRLPEFSDRNPGVQQRAQEEIDRIASGSAGQSVALESMERLPEFSDRSSLPYIEAVWRFLDDKGGLTDDLPAYAFGFGRMSLAYLAGALVLAAAVLWKGVETR
2	Apoptosis activation factor 1	MDAKARNCLLQHREALEKDIKTSYIMDHMISDGFLTISEEEKVRNEPTQQQRAAMLIKMILKKDNDSYVSFYNALLHEGYKDLAALLHDGIPVVSSSSGKDSVSGITSYVRTVLCEGGVPQRPVVFVTRKKLVNAIQQKLSKLKGEPGWVTIHGMAGCGKSVLAAEAVRDHSLLEGCFPGGVHWVSVGKQDKSGLLMKLQNLCTRLDQDESFSQRLPLNIEAKDRLRILMLRKHPRSLLILDDVWDSWVLKAFDSQCQILLTTRDKSVTDSVMGPKYVV PVESSLGKEK GLEILSLFVNMKKADLPEQA HSIIKECKGS LE
3	Caspases 8	DATAKIRLVRHSLDSVDPTPRPRSHPQAAPQPQAHTPTASVPSRRRPSTPQAPPPPSMVSVDSPRRPSTPQAPPPPSMVSVDSPRMNASGKASAVASNVHAGPAAAGAMSFGWLGPRLSFGSPR
4	CREB3 regulatory factor	MLSATPLYGNVHSWMNSERVRMCGASEDRKILVNDGDASKARLELREENPLNHNVVDASTAHRIDGLAALSMDRTGLIREGLRVPGNIVYSSLCGLGSEKGREAATSTLGGLGFSSERNPEMQFKPNTPETVEASAVSGKPPNGFSAIYKTPPGIQKSAVATAEALGLDRPASDKQSPLNINGASYLRLPWVNPYMEGATPAIYPFLDSPNKYSLNMYKALLPQQSYSLAQPLYSPVCTNGERFLYLPPPHYVGPHIPSSLASPMRLSTPSASPAIPPLVHCADKFYGSSVCEPDDESGYDVLANPPGPEDQDDDDDAYSDVFEFEFSETPLLPCYNIQVSVAQGPRNWLLLSDVLKKLKMSSRIFRCNFPNVEIVTIAEAEFYRQVSASLLFSCSKDLEAFNPESKELLDLVEFTNEIQTLLGSSVEWLHPSDLASDNYW
5	BCL-6 corepressor	MLSATPLYGNVHSWMNSERVRMCGASEDRKILVNDGDASKARLELREENPLNHNVVDASTAHRIDGLAALSMDRTGLIREGLRVPGNIVYSSLCGLGSEKAGGKKQAQPSCAPAPLLPCYNIQVSVAQGPRNWLLLSDVLKKLKMSSRIFRCNFPNVEIVTIAEAEFYRQVSASLLFSCSKDLEAFNPESKELLDLVEFTNEIQTLLGSSVEWL HPSDLASDNY W
6	Protein BTG2	MSHGKGTDMLPEIAAAVGFLSSLLRTRGCVSEQRLKVFSGALQEALTEHYKHHWFPEKPSKGSGYRCIRINHKMDPIISRVASQIGLSQPQLHQLLPSELTLWVDPYEVSYRIGEDGSICVLYEEAPLAASCGLLTCKNQVLLGRSSPSK NYVMAVSS
7	Retinoblastoma	MPSGGDQSPPPPPPPPAAAASDEEEEDDGEAEDAAPPAESPTPQIQQRFDELCSRLNMDEAARAEAWDSYRSMSESYTLEGNDLHWLACALYVACRKSVPTVSKGTVEGNYVSLTRILKCSEQSLIEFFNKMKKWEDMANRTSRDSSPVMRSSSTLPVPQPSSAPPTPTRLTGANSDMEEEERGDLIQFYNNIYIKQIKTFAMKYSQANMDAPPLSPYPFVRTGSPRRIQLSQNHPVYISPHKNETMLSPREKIFYYFSNSPSKRLREINSMIRTGETPTKKRGILLEDGS ESPAKRICPE NHSALLRRLQ DVANDRGSH

## Data Availability

Data is contained within the article. The data presented in this study are available in [Solid-State Fermentation with Aspergillus niger GH1 to Enhance Polyphenolic Content and Antioxidative Activity of Castilla Rose (Purshia plicata)]. Data citation: De León-Medina JC, Sepúlveda L, Morlett-Chávez J, Meléndez-Renteria P, Zugasti-Cruz A, Ascacio-Valdés J, Aguilar Cristóbal N. 2020. Solid-State Fermentation with Aspergillus niger GH1 to Enhance Polyphenolic Content and Antioxidative Activity of Castilla Rose (Purshia plicata. Persistent identifier doi: 10.3390/plants9111518.
